# Calculate excess mortality during heatwaves using Hilbert-Huang transform algorithm

**DOI:** 10.1186/1471-2288-14-35

**Published:** 2014-03-04

**Authors:** Gang Xie, Yuming Guo, Shilu Tong, Lin Ma

**Affiliations:** 1 CRC for Infrastructure and Engineering Asset Management (CIEAM), Science and Engineering Faculty, Queensland University of Technology, Brisbane, Australia; 2 Department of Epidemiology and Biostatistics, School of Population Health, The University of Queensland, Brisbane, Australia; 3 School of Public Health, Queensland University of Technology, Brisbane, Australia

## Abstract

**Background:**

Heatwaves could cause the population excess death numbers to be ranged from tens to thousands within a couple of weeks in a local area. An excess mortality due to a special event (e.g., a heatwave or an epidemic outbreak) is estimated by subtracting the mortality figure under ‘normal’ conditions from the historical daily mortality records. The calculation of the excess mortality is a scientific challenge because of the stochastic temporal pattern of the daily mortality data which is characterised by (a) the long-term changing mean levels (i.e., non-stationarity); (b) the non-linear temperature-mortality association. The Hilbert-Huang Transform (HHT) algorithm is a novel method originally developed for analysing the non-linear and non-stationary time series data in the field of signal processing, however, it has not been applied in public health research. This paper aimed to demonstrate the applicability and strength of the HHT algorithm in analysing health data.

**Methods:**

Special R functions were developed to implement the HHT algorithm to decompose the daily mortality time series into trend and non-trend components in terms of the underlying physical mechanism. The excess mortality is calculated directly from the resulting non-trend component series.

**Results:**

The Brisbane (Queensland, Australia) and the Chicago (United States) daily mortality time series data were utilized for calculating the excess mortality associated with heatwaves. The HHT algorithm estimated 62 excess deaths related to the February 2004 Brisbane heatwave. To calculate the excess mortality associated with the July 1995 Chicago heatwave, the HHT algorithm needed to handle the mode mixing issue. The HHT algorithm estimated 510 excess deaths for the 1995 Chicago heatwave event. To exemplify potential applications, the HHT decomposition results were used as the input data for a subsequent regression analysis, using the Brisbane data, to investigate the association between excess mortality and different risk factors.

**Conclusions:**

The HHT algorithm is a novel and powerful analytical tool in time series data analysis. It has a real potential to have a wide range of applications in public health research because of its ability to decompose a nonlinear and non-stationary time series into trend and non-trend components consistently and efficiently.

## Background

Historical records showed that heatwaves could cause the population excess death numbers to be ranged from tens to thousands within a couple of weeks in a local area [1]. For example, 465 heat-related deaths were recorded during the July 1995 Chicago (17-day) heatwave period [2] and the August 2003 heatwave caused more than 20000 excess deaths in Southern Europe counties [1]. Therefore, heatwaves can have big impacts on public health. An excess mortality due to a special event (e.g., a heatwave or an epidemic outbreak) is estimated by subtracting the expected mortality (i.e., the mortality figure under ‘normal’ conditions) from the historical daily mortality time series records. The calculation of the excess mortality is a scientific challenge because of the stochastic temporal pattern of the daily mortality time series data which is characterised by (a) the long-term changing mean levels (i.e., non-stationary); (b) the non-linear temperature-mortality association.

A consistent and efficient algorithm is essential to decompose a daily mortality time series into different trend series (i.e., the long-term trend, seasonal variation, and other unknown trends) and non-trend series (i.e., the short term fluctuations, or excess/deficit death counts due to extreme events). However, commonly accepted algorithms for time series decomposition in public health studies suffer the weakness of an arbitrary choice of smoothing parameters and/or are poor in adapting to local features [3,4]. For example, *moving average*, *differencing*, and various *smoothing splines* are widely used to analyse daily mortality time series data [1]. These existing algorithms are either shackled by spurious harmonics (signal processing approach) or weakened by subjective choice of smoothing parameter (statistical approach) [3-5]. These algorithms always put the mathematical correctness and tractability before the true representation of the underlying physical mechanisms. The Hilbert-Huang Transform (HHT) algorithm was introduced to analyse daily mortality time series data as HHT is probably the best available tool for analysing nonlinear and non-stationary time series [4,5].

The HHT algorithm was originally designed in 1995, under the name of EMD and HSA (Empirical Mode Decomposition and Hilbert Spectral Analysis), specifically to study water surface wave evolution [6]. Huang et al. [5] is commonly known as the original HHT algorithm article. The HHT algorithm is unique and different from other existing algorithms, because it is truly an adaptive time-frequency analysis. HHT reveals the true physical meanings in many of time series analyses [4,7]. It has been successfully used in various fields particularly in engineering and geophysical studies [4,6]. However, HHT is relatively new and has not been included as a standard statistical data analysis tool. To our knowledge, the HHT algorithm has not been applied in public health research.

This paper aimed to demonstrate the applicability and strength of the HHT algorithm in public health research. To illustrate the utilization of the HHT algorithm, the Brisbane daily mortality data [8] and the Chicago daily mortality data from the National Morbidity, Mortality, and Air Pollution Study (NMMAPS) were used to calculate the excess mortality due to heatwaves. The HHT decomposition results were further used as the input data for a subsequent regression analysis to investigate the association between excess mortality and different risk factors such as temperature, ozone, and particulate matter. Through the application examples, the procedure of applying HHT to decompose the daily mortality series was explained step by step. The HHT applications in this paper were implemented using special R functions [9] developed from the latest HHT research results. More technical details about the HHT algorithm and the R codes are provided in the Additional file 1.

## Methods

### Example data

As mentioned above, two data sets were employed in this study: 1) the Brisbane daily mortality and meteorological data; 2) the Chicago daily mortality data.

The Brisbane data set had been used to calculate excess deaths during the February 2004 heatwave by Tong et al. in 2010 [8]. Daily time series data on non-external deaths were obtained from the period 01/07/1996 to 30/06/2004. The two identified heatwave events were in 2000 and 2004 summer seasons, respectively. Previous research showed that daily maximum temperature is one of the best predictors of daily mortality [10], and was therefore used as the primary risk factor to perform the regression analysis. Air pollution data for the same period were also used and included daily average concentrations of PM_10_ (particulate matter with equivalent diameters less than 10 μm) and O_3_ (ozone).

The Chicago daily mortality data were extracted from the well-known NMMAPS dataset for the period 01/01/1995 to 30/06/2000. There were 4780 classified heat-related deaths in the U.S. over the period 1979 to 2002, among which 465 deaths were recorded due to the 1995 Chicago heatwave (11–27 July) [2,11]. This formed a reference for comparison of the HHT algorithm result with previous findings.

### HHT algorithm

The key part of the HHT algorithm is the Empirical Model Decomposition (EMD) process with which any complex time series data set can be decomposed into a finite and often small number of components, called Intrinsic Mode Function (IMF). A time series is called an IMF if it satisfies the following two criteria: (1) The number of local extreme values (maxima or minima) of the time series and the number of its zero-crossings must either be equal or differ by at most one; (2) At any time, the mean value of the upper envelope determined by the local maxima and the lower envelope determined by the local minima is zero [4]. In the real world, IMFs can lead to physically meaningful definitions of instantaneous frequency and instantaneous amplitude. In this sense, HHT provides a more physically meaningful time-frequency-energy description of the original time series. Technically, the IMFs are generated through a sifting process. Interested readers may refer to the references [4]–[6,12] and the Additional file 1 for more details.

Denote a time series by *X(t)*. By applying the EMD process, the original time series can be decomposed as

(1) $$X\left(t\right)=\sum_{j=1}^{N}c_{j}+r_{N},$$

where *c*_*j*_ is the *j*th IMF and *r*_*N*_ is the residual series after *N* IMFs are extracted.

Once the IMFs are generated, a significance test can be applied to identify the trend components (i.e., which IMFs should be treated as trend components) from those non-trend components. There are two approaches to construct the significance tests: one is developed by Wu and Huang using Matlab programs [7,13,14], following an analytic approach resorting to the central limit theorem; the other is based on research conducted by Flandrin et al. [15], following a Monte Carlo simulation approach. Both approaches were consistent in the tested results [13]. This study followed Flandrin's approach and developed the significant test procedure as detailed below.

**Figure 1:**
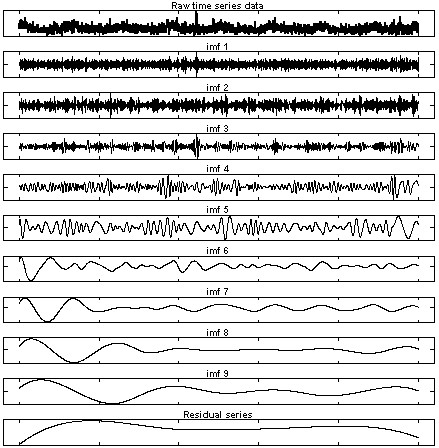
IMFs decomposed from Brisbane daily mortality time series (eight years period).

In general, the non-trend components (or noise/random components as usually termed in signal processing study) are found among the low-order (i.e., high-frequency) IMFs, which are intuitively clear as shown in Figure 1 (row 2 to row 9). Let *E*_*k*_ denote the energy level for the *k*th IMF generated from the EMD process. According to the method developed by Wu and Huang [13] and Flandrin et al. [15], the observed/estimated IMF energy level can be calculated by

(2) $${\hat{E}}_{k}=\sum_{i=1}^{n}{\left[d_{k}\left(i\right)\right]}^{2},\;k=1,2,\ldots ,N,$$

where *d*_*k*_(*i*) is the *i*th element value of the *k*th IMF; *n* is the data length or sample size of the original time series; and *N* is the total number of IMFs, e.g., *N* = 9 in Figure 1. Because of the Hilbert transform, the squared amplitudes (i.e., [*d*_*k*_(*i*)]*²*) represent the energy levels of each IMF series [5,6].

**Figure 2:**
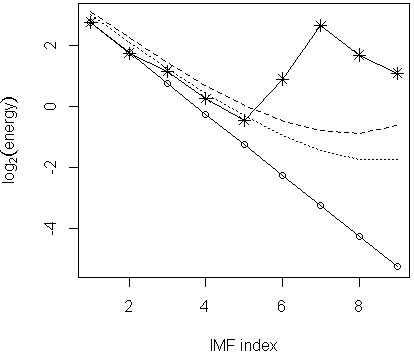
**Significance test to separate the trend IMFs from non-trend IMFs (Brisbane data).** The solid line connecting the circle points represents the white noise energy levels for each IMF; solid line segments connecting star points is the observed IMF energy level curve. Dotted line is the approximate 95% confidence band (upper limit); dashed line is the 99% confidence band (upper limit).

For signals involving Gaussian white noise, the energies of the non-trend component IMFs can be approximated as [15]

(3) $${\hat{W}}_{H}\left[k\right]=\frac{{\hat{E}}_{1}}{0.719}\times 2.01^{-k},\;k=2,3,\ldots ,N,$$

where $\;{\hat{E}}_{1}$ can be obtained from Equation (2). As a result, the IMF energies, ${\hat{W}}_{H}\left[k\right]$, for each order *k*, should decrease linearly when displayed on a semi-log plot, e.g., $\log_{2}\left\{{\hat{W}}_{H}\left[k\right]\right\}$ versus *k* as shown by the solid straight line (connecting circle points) in Figure 2. Any IMFs with which their $\log_{2}\left[{\hat{E}}_{k}\right]$ values deviate significantly from the white noise (decreasing straight) energy line towards the top-right corner should be identified as trend IMFs [7,15].

In the case that the non-trend IMFs have significant serial correlations, the significance test can be generalized as follows: if a well-fitted least-square line is obtained from the first few low order IMFs and this line is extrapolated to the full range of the order index, any higher order (i.e., lower frequency) IMFs which significantly deviate from this line towards the top-right corner should be identified as trend IMFs. Those lower order (i.e., high-frequency) IMFs from which the well-fitted least-square line is formed are the non-trend IMFs [15].

As shown in Figure 2, the approximate 95% (dotted lines) and 99% (dashed lines) confidence bands can be obtained for the Gaussian white noise series energy line. For the 95% confidence band, the plotting ordinates, *upper*95, are given by [15]

$$\text{upper}95\left[k\right]=\log_{2}\left\{{\hat{W}}_{H}\left[k\right]\right\}+2^{0.474k-2.449},$$

for *k* = 2, 3, …, *N*. For the 99% confidence band, the plotting ordinates, *upper*99, are given by [15]

$$\text{upper}99\left[k\right]=\log_{2}\left\{{\hat{W}}_{H}\left[k\right]\right\}+2^{0.460k-1.919},$$

for *k* = 2, 3, …, *N*. Therefore, based on the significance test result, the original time series *X(t)* can be decompose as

(4) $$X\left(t\right)=\sum_{i=1}^{m}c_{i}+\sum_{j=m+1}^{N}c_{j}+r_{N},$$

(for 1 < *m* < *N*) where $\sum_{i=1}^{m}c_{i}$ is the ‘detail’ or the non-trend components of *X(t)* and $\sum_{j=m+1}^{N}c_{j}+r_{N}$ is the trend components of *X(t)*.

**Figure 3:**
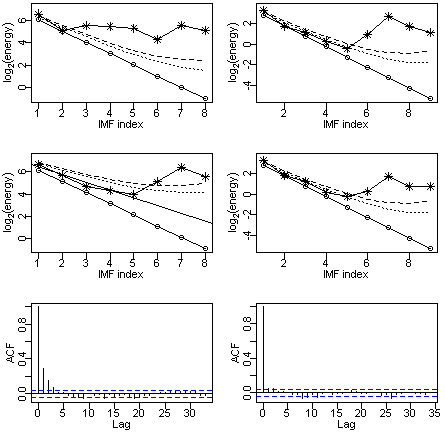
**Significance test to separate trend IMFs from non-trend IMFs and standard autocorrelation function (ACF) plot.** The solid line without circle points (middle-left plot) is the fractional white noise energy level line. The dashed lines in the ACF plots (bottom row) are the approximate 95% confidence bands. Dotted line is the approximate 95% confidence band (upper limit); dashed line is the 99% confidence band (upper limit).

If a significant serial correlation exists in the identified non-trend series, the *upper*95[k] and *upper*99[k] will shift up (if positively correlated) or down (if negatively correlated) characterised by the best-fit least square line as shown in Figure 3 (middle-left plot). This is the fractional Gaussian noise series case which includes the Gaussian white noise series as a special case [15].

The ‘detail’ part of the *X(t)* is needed for calculating the excess mortality while the trend part is needed for calculating the relative risk in a regression analysis.

**Figure 4:**
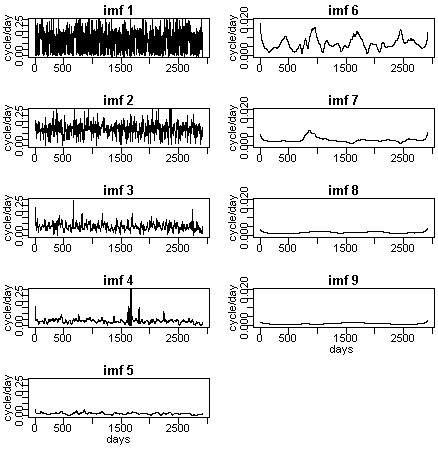
Instantaneous frequency plots for each IMFs.

If needed, the instantaneous frequencies can be calculated for each IMF as shown in Figure 4. The definition of the instantaneous frequencies of a time series and detailed calculations may be found in the references [4,5,7].

The EMD process can fail with the presence of a ‘mode mixing’ issue. The ‘mode mixing’ issue is defined as a single IMF either consisting signals of widely disparate scales, or a signal of a similar scale residing in different IMF series. Mode mixing is often a consequence of signal intermittency [16]. The analysis of the Chicago data encountered the mode mixing issue, as shown in the left column plots of Figure 3. Using the ensemble EMD (EEMD) process is the solution to a mode mixing issue. The principle of the EEMD process is that the added white noise would populate the whole time-frequency space uniformly with the constituting components of different scales. The EEMD process defines the true IMFs as the mean of an ensemble of trials, each consisting of the signal plus a white noise of finite amplitude (usually we choose 0.1 standard deviation of the original time series) [4,13,16]. For example, the EEMD process can be applied by running 100 times of EMD decomposition processes with an added white noise of 0.1 standard deviation of the original time series. The average of the 100 EMD results is then taken as the final decomposition results of an EEMD process.The significance tests are applied to the IMFs generated from the EMD and EEMD processes, respectively. If they agree, such as the case of Brisbane data analysis (plots in the right column of Figure 3), the EMD process is sufficient for generating IMFs; otherwise, the EEMD process is needed.

Once the original time series is decomposed into trend and non-trend IMFs, calculation of the excess mortality or other data analysis procedures (e.g., a subsequent regression analysis) can be performed using the resulting IMFs as the input.

## Results

### Brisbane data analysis

The EMD process was applied to decompose the Brisbane daily mortality data and the results were shown in Figure 1. The top plot showed the original time series, followed by the IMF series (IMF 1 to IMF 9), and the bottom plot represented the ‘residual’ series. Since the residual series was obtained by removing all the higher frequency components (IMFs) from the original time series, it was always one of the trend components. The x-axis measured the time in days starting from 01/07/1996 and the y-axis measured the total or decomposed daily mortality. The (element wise) sum of the series of IMFs 1 to 9 and the ‘residual’ series recovered the original series (top plot) as defined by Equation (1).

Based on Figure 2 and the significance test rule specified in the previous section, IMFs 1 to 5 were identified to form the non-trend part of the daily mortality time series. The non-trend series provided the excess mortality estimates for each day of the observation period. Therefore, if 07/02/2004 to 26/02/2004 (20 days) was specified as the heatwave period, the excess mortality due to this heatwave was simply the sum of the resulting non-trend series over this 20-day period. The estimated excess mortality total was 62 (rounded to integer). Tong et al. [8] obtained a 95% confidence interval estimation (11, 138) of the excess deaths from a fitted Generalised Additive Model (GAM) [17]. Based on Table 1, it was trivial to find out that it was those peak heatwave days made the dominant excess mortality contribution, that was, 52 deaths in six days. In Table 1, notation MaxT represented the daily maximum temperature; those ExcessDeath values were obtained directly from the resulting non-trend series.

**Table 1:** Excess mortality over the period 18–23 February, 2004, Brisbane

**Date**	**18**	**19**	**20**	**21**	**22**	**23**	
**MaxT (°C)**	30.5	32.1	32.0	35.2	40.2	31.6	**Sum**
**ExcessDeath**	5.7	6.7	7.6	-0.4	20.6	11.6	**51.8**

The strength of the HHT algorithm is that the decomposed IMFs capture the local features of a time series (i.e., the instantaneous frequency and instantaneous amplitude) better than any other available algorithms [4,5]. Figure 4 showed the instantaneous frequency plots for all nine IMFs. For comparison, those IMFs identified as non-trend components (i.e., IMFs 1 to 5) were displayed in the left column; trend component IMFs were displayed on the right. The y-axis represented the instantaneous frequency in terms of cycles per day. Therefore, the instantaneous cycle length (i.e., periodic pattern) could be calculated by taking the inverse of the instantaneous frequency. This detailed frequency pattern information is important because it helps us to understand the underlying physical processes better.

For estimation of the average period (i.e., the average cycle length in days for daily time series data), as proposed in [13], the calculation formula is given as

$$\text{average}\;\text{period}\left(k\right)=n/\text{number}\;\text{of}\;\text{peaks}\left(k\right),$$

where the average period(*k*) is the average period in days of the *k*th IMF; *n* is the data length or sample size of the original time series; number of peaks(*k*) is the number of the local maxima points of the *k*th IMF for *k* = 1, 2, …, *N*. With the Brisbane data, we had *n* = 2922 and *N* = 9. Table 2 showed the estimated average period in days for each IMF of the Brisbane data. Note that, since the EMD process is essentially a dyadic filter bank, it decomposes the original time series in a manner that the average periods are approximately doubling the neighbouring IMFs [4,6].

**Table 2:** Average period of each IMF (in days)

**IMF1**	**IMF2**	**IMF3**	**IMF4**	**IMF5**	**IMF6**	**IMF7**	**IMF8**	**IMF9**
2.9	6.2	12.8	25.6	54.1	146.1	292.2	487.0	730.5

### Chicago data analysis

We applied the EMD process to the Chicago data (graphic output not shown) and performed the significance test as shown in the top-left plot of Figure 3. The plot indicated that only IMFs 1 and 2 should be treated as the non-trend components. In the original time series plot (not shown), a huge spike appeared around July 1995 which may cause the mode mixing problem. We then applied EEMD to the Chicago data and performed the significance test accordingly. The result was shown in the middle-left plot of Figure 3. Comparing the results obtained from EMD and EEMD (i.e., top-left plot versus middle-left plot), we concluded that the mode mixing issue could not be ignored and the EEMD process was necessary. By applying the generalized significance test rule, the IMFs 1 to 5 were identified as the non-trend components (middle-left plot). In Figure 3, the significance test results for the Brisbane data (plots in the right column) were included for comparison: both the EMD and EEMD generated IMFs test results (top and middle plots in the right column of Figure 3) seemed to be in agreement with each other. Therefore, the EMD decomposition was sufficient for Brisbane data.

With the Chicago data, the non-trend components energy line was deviated (upwards) from the white noise energy line due to its significant serial correlation. The bottom row plots compared the autocorrelation function curves of the Chicago data with the Brisbane data. The Chicago data (bottom-left plot) exhibited a significant serial correlation – lags 1 and 2 serial correlations were well beyond the 95% confidence band. This explained the mismatch between the Chicago data IMF energy line and the white noise energy line.

Once the non-trend IMFs were determined, given a specified heatwave period, the calculation of the excess mortality of the Chicago data became straightforward. The HHT algorithm estimated the excess mortality over the period 11–18 July 1995 to be 510. According to the Mortality and Morbidity Weekly Report (MMWR), a total of 465 deaths were identified as heat-related in Chicago during 11–27 July [2]. The MMWR is the official source to publish the heat-related death figures reported by the Cook County Medical Examiner’s Office (CCMEO) during heatwaves [2].

### More on Brisbane data analysis

Relative risk in natural logarithm scale, denoted by log_e_(RR), was used as the health outcome variable for the regression analysis. The log relative risk was calculated as (following the notation defined in Equation (4))

(5) $$\log_{e}\left(\text{RR}\right)=\log_{e}\frac{P_{\text{exposed}}}{P_{\text{non}-\text{exposed}}}={\text{log}}_{e}\frac{X\left(t\right)}{\sum_{i=6}^{9}c_{i}+r_{9}},$$

where *X(t)* was the original daily mortality series and $\sum_{i=6}^{9}c_{i}+r_{9}$ was the trend series obtained from the EMD process in the ‘Brisbane data analysis’ subsection. The trend series represented the daily mortality under ‘normal’ conditions.

Similar to the decomposition of the daily mortality data, the EMD process was applied to the daily maximum temperature series, denoted by maxT, to generate the IMFs. The maximum daily temperature data IMFs 1 to 5 were grouped to form the ‘non-trend’ part as it had the same frequency structure as the identified non-trend daily mortality series. The non-trend part of the daily maximum temperature, denoted by maxT_(non-trend)_, represented the daily maximum temperature anomaly. The association between the daily mortality and the maximum temperature was then investigated by fitting the simple ordinary linear regression models with the following settings. The fitted models were specified using the R code notation as given below:

Model 1 (heatwave period): lm(log_e_(RR) ~ maxT_(non-trend)_)

Model 2 (heatwave period): lm(log_e_(RR) ~ maxT)

Model 3 (summer season): lm(log_e_(RR) ~ maxT_(non-trend)_)

Model 4 (summer season): lm(log_e_(RR) ~ maxT)

where the ‘heatwave period’ referred to the 20-day period 7–26 July, 2004; the ‘summer season’ referred to the 91-day period from 01/12/2003 to 29/02/2004. The regression analysis results were given in Table 3.

**Table 3:** Regression analysis result (I)

**Model**	**Coefficient of maxT or maxT**_**(non-trend)**_	**p-value**	**Adjusted R**^**2**^	**2004(period)**
**1**	0.06553	0.0248	0.208	Heatwave (20 days)
**2**	0.06223	0.0390	0.172	Heatwave (20 days)
**3**	0.05705	<0.001	0.127	Summer (91 days)
**4**	0.05652	<0.001	0.165	Summer (91 days)

Table 3 showed that all four models were statistically significant at 0.05 level. For the association between the excess mortality and daily maximum temperature, the heatwave period had a stronger positive correlation (larger positive coefficient and higher adjusted R^2^ value) than the summer season period. Models 1 and 3 measured the association of excess mortality with the excess maximum temperature (i.e., anomaly) but models 2 and 4 measured the association of the excess mortality and maximum temperature (i.e., absolute temperature level). We found that it was the impact (R^2^: 0.208 versus 0.127) of the maxT anomaly that differed more than the impact (R^2^: 0.172 versus 0.165) of maxT itself between the heatwave period and the summer season as a whole.

Finally, we investigated the associations between log_e_(RR) and maxT, O_3_, and PM_10_ for summer seasons of 2000 and 2004. The summer season of 2000 was defined as a 91-day period from 01/12/1999 to 29/02/2000. We decided to compare these two summer seasons because there was a similar heatwave in Brisbane in 2000 as it was in 2004. We specified six models in R code format as given below among which models 1 to 4 referred to the year 2000 summer season and models 5 and 6 referred to year 2004 summer season. The regression analysis results for each model were given in Table 4.

**Table 4:** Regression analysis result (II)

**Model**	**Coef. of maxT**	**Coef. of PM10**	**Coef. of O3**	**p-value**	**Adjusted R**^**2**^	**Year (91 days)**
**1**	0.04766	-	-	0.0034	0.082	2000 (summer)
**2**	0.02315	0.03016	-	<0.001	0.215	2000 (summer)
**3**	0.03391	-	0.01567	0.0022	0.110	2000 (summer)
**4**	0.02094	0.02852	0.00404	<0.001	0.208	2000 (summer)
**5**	0.05652	-	-	<0.001	0.165	2004 (summer)
**6**	0.05225	0.00712	-	<0.001	0.166	2004 (summer)

Model 1 (2000 summer): lm(log_e_(RR) ~ maxT)

Model 2 (2000 summer): lm(log_e_(RR) ~ maxT + PM10)

Model 3 (2000 summer): lm(log_e_(RR) ~ maxT + O3)

Model 4 (2000 summer): lm(log_e_(RR) ~ maxT + PM10 + O3)

Model 5 (2004 summer): lm(log_e_(RR) ~ maxT)

Model 6 (2004 summer): lm(log_e_(RR) ~ maxT + PM10)

After testing different regression models, the following association relationships were found. For year 2000 summer season, PM_10_ had a very strong impact on the excess daily mortality, and therefore the daily maximum temperature became a less important risk factor in terms of the adjusted R^2^. Furthermore, there was a high correlation (Pearson correlation coefficient is 0.501) between PM_10_ and O_3_ during the year 2000 summer season. A further inclusion of O_3_ (i.e., Model 3 and Model 4) did not make a statistically significant difference. For year 2004 summer season, however, neither PM_10_ nor O_3_ had a significant impact on the excess mortality, i.e., maxT was the only significant risk factor among these three factors.

## Discussion

In the Chicago data analysis we encountered the mode mixing issue and the EEMD process was employed to generate IMFs. Unfortunately, the EEMD decomposition does not generate exact IMFs (hence Hilbert transform may not be applicable) and we need to perform the post processing treatment [16]. Wu and Huang proposed a general post processing procedure for EEMD generated IMF-like series. Details can be found on page 29 of the reference [16]. In the Chicago data analysis, a special R function postEEMD was developed to perform the post processing treatment. Because EEMD is a noise assisted method, the added white noise causes some random fluctuation of the decomposed results. To minimise the random fluctuation effect, we have run a batch of 20 EEMD and post treatment processes on the data. Ultimately, 20 sets of post processed EEMD IMFs were generated and we took the averages as the final IMFs for the Chicago data analysis. For the repeatability of the analysis results, we initiated the decomposition process by setting the random seed to 101 in R and the resulting estimated excess mortality total during the 1995 Chicago heatwave was 510.

**Figure 5:**
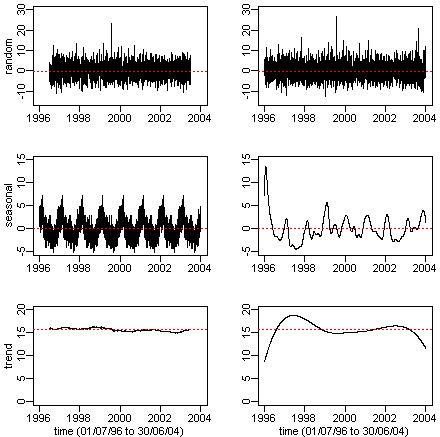
A comparison between the standard time series decomposition versus HHT decomposition.

Another concern is the stoppage rule. Different stopping rule can produce similar but not exactly the same IMFs. By setting the number of iterations of the sifting process to 10, recent researches suggested this stoppage rule to be the most optimal and it ensures the uniqueness of the generated IMFs [12,16]. As the founder of the EEMD process, Wu prepared a full set of Matlab programs [14] to implement HHT algorithm which are free available in [7]. These Matlab programs can perform the major HHT algorithm functions such as EMD, EEMD, generation of instantaneous frequency series, and significance testing. Our special R functions prepared for this study were essentially a translation of Wu's Matlab programs except for the significance test function which is our original work. The EEMD post processing functionality was also added in our R programs as mentioned before. The Additional file 1 contains more details of the functionalities, usage and discussions on our special R functions of the HHT algorithm.Figure 5 compared the standard time series decomposition with the HHT decomposition using the Brisbane daily mortality data. The standard time series decomposition employed the moving average (frequency = 365) algorithm to identify the trend series (bottom-left plot). The ‘seasonal’ series was defined as the average annual pattern of the sample data. Therefore, it showed a regular repeating cyclic pattern for each year (middle-left plot). After removing the ‘trend’ and ‘seasonal’ patterns from the original data, we obtained the ‘random’ series (top-left plot). Because of the end-point-effect of the moving average process, the information loss on both ends of the ‘trend’ and ‘random’ series was clearly seen (bottom-left and top-left plots). For the HHT decomposition, the ‘trend’ series (bottom-right plot) was obtained from the ‘residual series’ in Figure 1. The ‘seasonal’ series (middle-right plot) was the element-wise sum of IMFs 6 to 9 in Figure 1. The ‘random’ series (top-right plot) was obtained by summing up the IMFs 1 to 5 (Figure 1) as explained in the ‘Brisbane data analysis’ subsection.Figure 5 showed that the mean levels (dotted horizontal lines) of the corresponding series were the same for both algorithms. However, the frequency and amplitude modulations differed significantly. As we argued in the previous sections, the random part of the daily mortality (i.e., the non-trend series) contained all the excess mortality information. In the top row plots of Figure 5, both algorithms identified the highest (positive) spike for the year 2000 summer. A big positive spike represented the extraordinary magnitude of the excess mortality at a particular time point. Note that no information was available for the year 2004 summer from the standard time series algorithm (top-left plot), while the HHT algorithm clearly showed the second highest spike occurred in the year 2004 summer (top-right plot). Thus, the standard time series decomposition results could not be used for calculating the excess mortality related to the 2004 heatwave because of the end-effect information loss. Secondly, a repeating regular seasonal pattern was certainly too far from a true representation of the actual seasonal fluctuation pattern which was better represented by the HHT results. In the seasonal patterns (middle row plots), although both algorithms had identified nine peaks (corresponding to nine winter periods), the HHT algorithm (middle-right plot) revealed more local features, e.g., a big peak for the 1996 winter (we may want to investigate why). By comparison, the standard time series decomposed cyclic patterns (middle-left plot) failed to distinguish these local variations. Finally, the ‘trend’ pattern obtained from the standard time series decomposition (bottom-left plot) contained far less information (almost a horizontal line since that most local features were evened out by the moving averages) than that in the counterpart HHT plot. Through the qualitative comparison, Figure 5 showed us a typical example of the advantages of the HHT decomposition over the standard time series decomposition.

Depending on different research aims, researchers may give different interpretations to the same HHT decomposition result, hence may find different applications. For example, from the signal processing perspective, researchers may focus on the identified trend IMFs and consider these IMFs contain the ‘true’ signals of the underlying physical process; hence, the non-trend series is termed as ‘noise’ for containing no useful information. By contrast, in the calculation of excess mortality, we focused on the non-trend IMFs which contained exactly the information of our concerns. As with any other analysis algorithm, to its best, HHT can only fully reveal the information contained in the data. External information or expertise and subject knowledge are needed to better or correctly interpret and utilize the HHT analysis results.

## Conclusions

The HHT algorithm provides a useful and novel approach to analyse time series data in public health area. In this paper, we demonstrated how to use the HHT algorithm to calculate the excess mortality during heatwave days. It is obvious that these applications can be repeated for any special events which have significant impacts on the daily mortality or morbidity, e.g., the SARS outbreak in 2003 and the H1N1 bird-flu event in 2009 [18,19]. We have focused on illustrating the applicability and power of the HHT algorithm as a new analytical tool in public health research. We believe that public health researchers will find different and better ways to exploit the potential of the HHT algorithm. For example, the possible combination of the General Additive Model [17] with the HHT algorithm may produce more physically meaningful or robust forecasting results. The 2-dimension EMD algorithm, which is still at its development stage, provides the potential for spatial-temporal modelling applications in public health studies [4]. We will investigate these issues in our future research.

## Competing interests

Queensland University of Technology (Brisbane, Australia) will pay the article-processing charge.

## Authors’ contributions

GX carried out the methodology study, statistical analysis, and drafting of the manuscript. YG provided the raw data sets, participated in discussions on methodology and analysis results, and drafting of the manuscript. ST participated in discussions on comparison and interpretation of the analysis results, and drafting of the manuscript. LM participated in discussions on methodology and analysis results, and drafting of the manuscript. All authors read and approved the final manuscript.

## Pre-publication history

The pre-publication history for this paper can be accessed here:

http://www.biomedcentral.com/1471-2288/14/35/prepub

## Supplementary Material

Additional file 1 This document gives the details of functionalities, usage and discussions on our special R functions for implementation of the HHT algorithm. Click here for file
