# Supplementary material for: Calculate excess mortality during heatwaves using Hilbert-Huang transform algorithm
Source: BMC Med Res Methodol. 2014 Mar 4;14:35. doi: 10.1186/1471-2288-14-35 (PMC3946180; doi:10.1186/1471-2288-14-35)
Supplement: Additional file 1 — This document gives the details of functionalities, usage and discussions on our special R functions for implementation of the HHT algorithm. [file 1471-2288-14-35-S1.docx]

**Appendix** to article “Calculate excess mortality during heatwaves using Hilbert-Huang Transform algorithm”.

For basic reading and review of HHT algorithm, readers are referred to the references [1-4, 6].

For HHT algorithm analysis programs, two resources may be found useful: (1) the Matlab programs which perform most of the HHT algorithm functions available from [7], authored by Zhaohua Wu; (2) the R package ‘EMD’ authored by Donghoh Kim and Hee-Seok Oh (published on 29/10/2012). We decide not to use ‘EMD’ package directly for our analysis, because (1) no significance test functionality available; (2) no post processing functionality available; (3) different stoppage rule in implementation of the EMD process; (4) user manual is not very helpful.

In this study, we have prepared our own special R functions to implement the HHT algorithm using the above two resources as the major references.

There are three attached files as part of this appendix. The plain text file ‘HHT-R-code-XIE-Dec2012.txt’ contains all the special R functions for the implementation of the HHT algorithm. Using the Chicago data as an example, we prepared R codes for readers to reproduce those analysis results presented in our paper. These R codes with some explanation remarks alongside are stored in file ‘R-code-forExamples’. The third file is the ‘ChicagoData.rda’. Readers should start with the file ‘R-code-forExamples’ and follow the instructions therein. The names, functionalities, usage of the special R functions, and discussions are given as follows.

**EEMDR**: is essentially a R translation of Matlab [5] program ‘eemd’ available from reference [7]; the major difference between EEMDR and eemd is in its subroutine function ‘extrema’. The function **extrema** called by EEMDR is a verbatim copy of the ‘extrema’ function from the R package ‘EMD’ authored by Donghoh Kim and Hee-Seok Oh. Function ‘extrema’ returns the following information: positions of minima and maxima, number of extrema, and number of zero crossing. Depending on specification of the input arguments, users can decompose the input time series by EMD process or EEMD process. EEMDR gives both the numeric output and graphic output of the generated IMFs.

Example 1: imf.emd = EEMDR(dailyD)

where ‘dailyD’ is the original time series data of daily mortality. R command of Example 1 performs an EMD decomposition on the input data time series ‘dailyD’. The graphic output of the decomposed IMFs is shown on screen and the numeric results are stored as an R object ‘imf.emd’.

Example 2: imf.eemd = EEMDR(dailyD,0.1, 100)

R command of Example 2 performs an EEMD decomposition on the input data time series ‘dailyD’. The numeric results of the generated IMFs are stored in the resulting R object ‘imf.eemd’. The second argument 0.1 specifies the fraction of the standard deviation of the input data with which the added noise series are constructed; the third argument 100 specifies the number of ensemble samples. The resulting IMF-like components are the averages of the 100 samples decomposition results.

In general, the EMD process will generate no more than log_2_(n) – 1 IMFs. The end point effects and the stoppage rule are the two major concerns for the EEMDR function. Both issues are handled in the Matlab code function ‘eemd’ according to the research results given in reference [7]. In particular, the stoppage rule is to set the number of iteration to 10 for the sifting process in determining the IMFs. In EEMDR, the end effects of EMD are treated explicitly according to the PPT document 'End Effects of EMD: an unsolved, and perhaps, unsolvable problem' presented in [7]. Therefore, EEMDR is an R realisation of the Matlab code in these respects. This stoppage rule makes the generated IMFs unique and empirical evidences showed that this stoppage rule is an optimal one [4].

**EEMDR1**: is a simplified version of EEMDR function. EEMDR1 acts as a subroutine function to be called by function ‘postEEMD’. EEMDR1 generates the post processed IMFs from EEMD process.

**postEEMD**: a function which generates the post processed IMFs. There is no counterpart Matlab function in [7].

Example 3: set.seed(101); imf.eemdp = postEEMD(dailyD, 0.1, 60)

R command of Example 3 performs a post treatment of EEMD decomposition results on the input data time series ‘dailyD’. The second and the third arguments have the same meanings as explained in Example 2. In the post processing step, the EEMD generated 'IMFs' are used as the input series to go through a final EMD process to generate the IMFs for subsequent analysis.

Unfortunately, the decomposed results by EEMD process do not guarantee they are the true IMFs. We need to do the post processing treatment on the EEMD generated IMF-like components. Note that the EEMD results are subject to the random fluctuations due to different random seeds involved in the added noise series generation process. Details of post processing treatment on EEMD can be found in [3] page 29.

**testimf**: This is a significance test function based on the average energy of IMFs to distinguish those trend IMFs from the "random/noise" (i.e. non-trend) IMFs. This function is of our original work by implementing the theoretic results given in [8].

Example 4: postmodes = imf.eemdp$postmode[,2:9]; testimf(postmodes)

testimf(postmodes, nfit=3, wnoise=0)

testimf(postmodes, nfit=4, wnoise=0)

The first R command line of Example 4 extracts the IMFs values and then perform the significance test; The second R command line checks what happens if we assume the White noise assumption does not hold (default value wnoise=1) and if assuming that the first three IMFs are the non-trend components (nfit=3); The third R command line checks if the first four IMFs are the non-trend components.

Wu [7] also provided a significance test function, ‘significance’ (need to call a subroutine function ‘dist_value’), in Matlab code. Based on our experience, Wu's Matlab program is equivalent to ours if the non-trend IMFs are close to white noise series. If the non-trend IMFs have significant serial correlation (i.e. deviate from Gaussian white noise series), according to Flandrin's approach [8], their log energies should still follow a linear pattern only with different gradient due to the Hurst exponent not equal to 0.5. We are not sure whether Wu's significance test is valid to have the same generalization.

**ifndqR**: This is a function to calculate instantaneous frequency based on HHT algorithm. This R function is a translation of the Matlab [5] code function ‘ifndq’ written by Zhaohua Wu [7].

Example 5: imf.emd = EEMDR(dailyD); ifndq.df = ifndqR(imf.emd$allmode[,2],1)

omega1 = ifndq.df$omega

The first line of the R command of Example 5 generates the IMFs and then extract the IMF 1 values and the calculated instantaneous frequency values for IMF 1 are stored in object ‘ifndq.df’; The second line of the R command extracts the instantaneous frequency values of IMF 1 into object ‘omega1’. Subsequently, omega1 can be plotted etc.

Although HHT can only generate the approximated IMFs, the approximation is accurate enough to produce practically useful/meaningful results [2,4]. HHT, although, still an empirical algorithm, has been proved to be the best available tool in analyzing the nonlinear and non-stationary time series.

**References:**

1. Huang NE, Shen Z, Long SR, et al. The empirical mode decomposition and the Hilbert spectrum for nonlinear and non-stationary time series analysis. Proceedings of the Royal Society London, Series A. 1998;454:903-995.
2. Huang NE, Wu Z. A review on Hilbert-Huang Transform: method and its applications to geophysical studies. Review of Geophysics. 2008;46:RG2006.
3. Wu Z, Huang NE. Ensemble Empirical Mode Decomposition: A Noise-Assisted Data Analysis Method. Advances in Adaptive Data Analysis. 2009;1:No.1,1-41.
4. Wang G, Chen X, Qiao F. On Intrinsic Mode Function. Advances in Adaptive Data Analysis. 2010;2:No.3,277-293.
5. MATLAB 2011a. MathWorks. Natick, Massachusetts, U.S.A. 2011.
6. Hilbert-Huang Transform. Available at: <http://www.scholarpidia.org/article/Hilbert-Huang_transform>. Accessed 15 September 2012.
7. Tutorial for the HHT MATLAB program. Available at: [http://rcada.ncu.edu.tw/research1. Accessed 11 October 2012](http://rcada.ncu.edu.tw/research1.%20Accessed%2011%20October%202012).
8. Flandrin P, Rilling F, Gongcalves P. EMD equivalent filter banks, from interpretation to applications. In book Hilbert-Huang Transform and Its Applications edited by Huang NE and Shen S.
